# Use of a Spinal Traction Device during Work Shift in Assembly Line Workers

**DOI:** 10.3390/ijerph18147708

**Published:** 2021-07-20

**Authors:** Juan Rabal-Pelay, Cristina Cimarras-Otal, Mónica Macia-Calvo, Carmen Laguna-Miranda, Ana Vanessa Bataller-Cervero

**Affiliations:** 1 Department of Physical Activity and Sports Sciences, Faculty of Health Science, Universidad San Jorge, 50830 Villanueva de Gállego, Spain; jrabal@usj.es (J.R.-P.); avbataller@usj.es (A.V.B.-C.); 2 Hospital MAZ, Avda. Academia General Militar 74, 500015 Zaragoza, Spain; monik_macia@hotmail.com; 3 BSH Electrodomésticos España S.A., Pol. Industrial Otallana, 50016 Zaragoza, Spain; Carmen.Laguna@BSHG.com

**Keywords:** low back pain, spinal sagittal alignment, spinal shrinkage, spinal traction, manufacturing workers, work break

## Abstract

Increasing back discomfort and spinal shrinkage during the workday is a problem that affects assembly line workers. The aim of this research was to analyze the effect of a spinal traction system on discomfort, spinal shrinkage, and spinal sagittal alignment in assembly line workers, who are in prolonged standing conditions during a workday. A total of 16 asymptomatic males were recruited to assess spinal shrinkage, spinal sagittal alignment, and back discomfort during the workday. The measurement was carried out in two days of work, a normal day, and the other using a spinal traction device utilized in two breaks during the workday. Assembly line workers lost height significantly on both control and intervention days. No differences were found between days. No changes were found in spinal sagittal alignment on the control day. Lumbar lordosis angle increased significantly at the end of the intervention day. The use of a spinal traction device during the workday in two breaks time did not significantly reduce the spinal shrinkage of healthy workers. Lumbar lordosis angle increased significantly at the end of the spinal traction intervention day. Prospective studies would be necessary to clarify the possible benefits of the traction device.

## 1. Introduction

Low back pain is one of the most common health complaints in industrially developed countries and the most frequently occurring occupational health problem. This issue increases work absenteeism and causes large costs in companies and the public health system [1,2].

Many workers are required to stand for long periods of time without being able to walk or sit during the work shift. Prolonged standing at work has been shown to be associated with low back pain and musculoskeletal discomfort [3,4,5]. Prolonged standing implies a load for the spine that is translated into a significant loss of height known as shrinkage [6,7,8].

The lumbar spine in upright standing is lordotic or concave posteriorly, thought to facilitate energy conservation during standing tasks [9]. Larger lumbar lordosis may be a risk factor for low back pain development during prolonged periods of standing [10]. Otherwise, people with low back pain tended to have attenuated lumbar lordosis, compared to asymptomatic people, especially due to disc pathology [11]. A previous study showed that workers on the assembly line, in prolonged standing work, suffer a loss of height, an increase of thoracic and lumbar curvatures, and low back discomfort [7]. Some research showed that spinal traction could improve back pain while maintaining lumbar lordotic curvature [12,13,14].

Different types of interventions in the workplace have been proposed to reduce and prevent back pain in workers: sit-stand workstations [15], exercise [16,17], stretching [18], footrests [19], breaks [20], insoles for shoes [21], etc. However, no studies have been found that addressed assembly line workers with spine traction intervention in prolonged standing conditions. The spine traction can be applied in many forms: monitored lumbar traction, auto-traction, manual traction, or gravitational traction using an inversion device [12,22].

The function of spinal traction is to generate a vertical stretch in the spine, relax the back muscles, and increase disc height [23,24]. Traction may work by separation of vertebral bodies, distraction, and gliding of facet joints, widening of the intervertebral foramen, straightening of the spinal curves, and stretching of the spinal musculature [24,25,26]. Spine traction and inversion traction devices seem to be effective in the reduction of low back pain and discomfort, [12,13] but the effects of their use in the prevention of back pain are not clear due to the variability of the studies carried out [22]. Therefore, further studies are needed to clarify its effect on low back pain and loss of height during the working hours of workers [3]. No previous studies have been found in relation to knowing how the use of spinal traction could affect the spinal sagittal alignment of people.

The aim of this study is to assess the effect of spinal traction on spinal shrinkage and spinal sagittal alignment in workers who are in prolonged standing conditions during a workday. A comparison between a worker’s control day and intervention day with a spinal traction device was evaluated. The initial hypothesis is that the use of a spinal traction device during two breaks in the work shift could reduce spinal shrinkage caused by the workday and modify the lumbar lordosis without increasing back discomfort.

## 2. Materials and Methods

### 2.1. Participants

In total, 16 volunteer participants (male) were recruited from an assembly line of a manufacturing company (38 ± 8 years, 80.4 ± 10.1 kg, 175.9 ± 3.7 cm). The recruitment was among workers who spend 8 h working in prolonged standing conditions. Machines and tools used in the assembly line are handled while standing. During the workday, workers have a 15 min break at 10:00 to have lunch and free time. Inclusion criteria included asymptomatic workers with Cornell’s musculoskeletal discomfort questionnaire equal to zero for the upper and low back area at the beginning of the day. Exclusion criteria included people diagnosed with scoliosis or low instability in the lumbar spine. The lumbar stability of the volunteers was tested with the passive lumbar extension test and lumbar instability in the prone test [27]. 

This research was an uncontrolled open trial. All participants were informed about the purpose and procedures of the study, as well as its possible risks and benefits. Every procedure was conducted in accordance with the principles of the World Medical Association´s Declaration of Helsinki. All participants signed an informed consent approved by the ethics committee of the region. The protocol was approved by the committee of ethics in research of the regional government [C.P.–C.I. PI18/087].

### 2.2. Outcome Measures and Method of Measurement 

**Figure 1:**
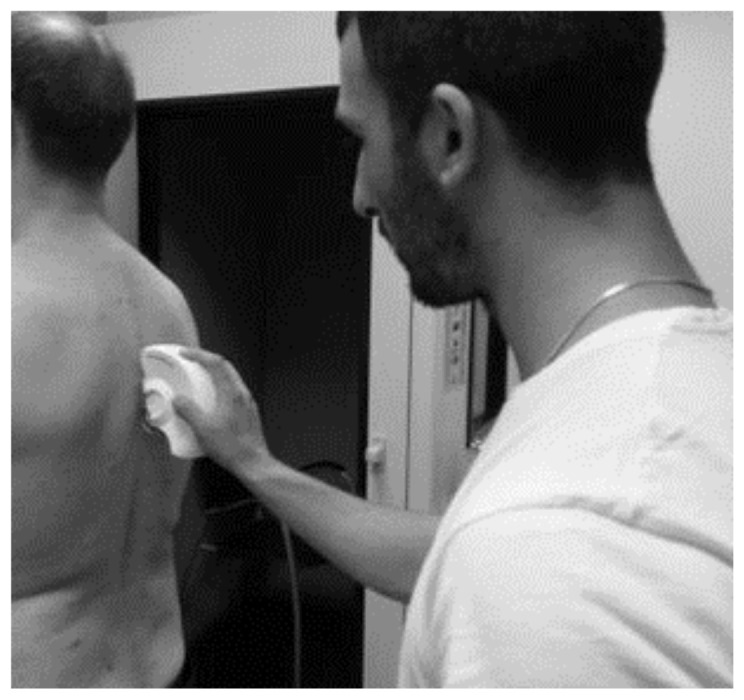
Thoracic and lumbar angles assessment with SpinalMouse^®^.

Height, body weight, spinal sagittal alignment, self-perceived upper and low back discomfort of the participants were assessed. Bodyweight outcome was evaluated to define the sample participants with a SECA^®^ calibrated digital scale (model 799, Seca Corp, Hanover, MD, USA) with a precision of 0.1 kg and a range of 2–200 kg. The angle of thoracic kyphosis and lumbar lordosis was used to assess the spinal sagittal alignment with SpinalMouse^®^ device (Idiag, Fehraltorf, Switzerland) (Figure 1) [7]. Height (cm) was measured using a SECA^®^ stadiometer (model 206, Seca Corp, Hanover, MD, USA) with a precision of 1 mm and a range of 130–210 cm. Spinal shrinkage was calculated with post–pre height values. The methodology and materials used to perform the measurement are detailed in Rabal-Pelay et al. [28].

Upper and lower back discomfort were assessed using Cornell´s musculoskeletal discomfort questionnaire for people standing. The questionnaire is used for knowing frequency, intensity, and interference caused by the discomfort of each body area in work tasks. In this study, the upper and lower back regions were included. This questionnaire has been translated and validated for use in the Spanish-speaking population [29]. 

### 2.3. Procedure

**Figure 2:**
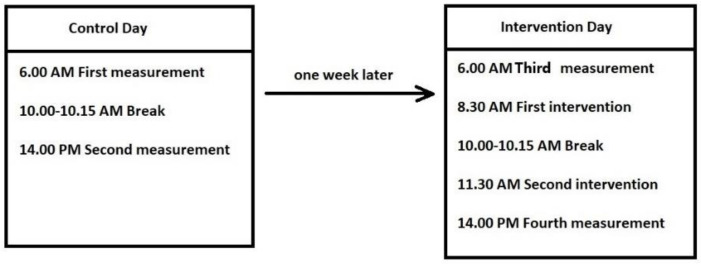
Control and intervention day timeline.

Data were collected in the medical service of the manufacturing company. Measurements were taken at 06:00 a.m. and 14:00 p.m., once the workday was completed. A week between control day and intervention day assessment was determined to measure in similar conditions (Figure 2). On control day, workers had a break of 15 min to rest, and have lunch and free time. On intervention day, in addition to the 15 min break, workers had two breaks during the workday for the intervention. The first intervention break was at 8:30 a.m. and the second at 11:30 a.m. Each additional break lasted 7 min, and a spinal decompressor device was used for 3 min in the presence of a physical coach. 

**Figure 3:**
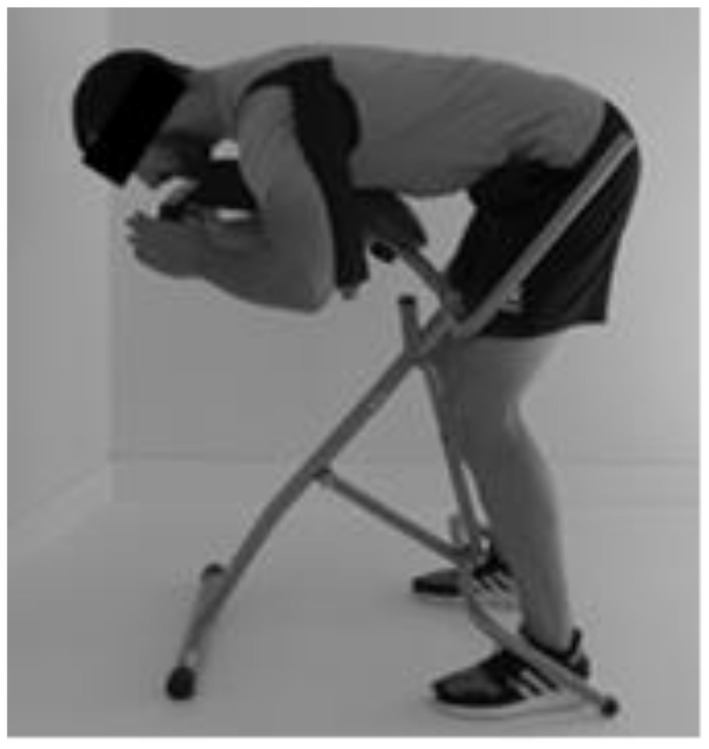
Use of the spinal decompressor to perform a self-traction of the thoracic and lumbar spine.

A spinal traction device of Quirumed Company was used for the intervention. Quirumed Spinal decompression device (Valencia, Spain) is indicated for people between 150 and 190 cm height. The device is adjustable for different heights (Figure 3). 

### 2.4. Statistical Analysis

Data are presented as mean and standard deviation. The Shapiro–Wilk test was applied to check the normal distribution of the variables. A repeated-measures ANOVA test was performed to study the differences between the four assessments. ANOVA was used for calculating main effects and a post hoc test was adjusted by Bonferroni. Inter-day effect size was calculated between differences in pre- and post-values for every variable. For eta-squared test, threshold values are interpreted as small (0.01), medium (0.06), and large effects (0.14) [30,31]. The level of significance was set at *p* < 0.05. The sample size was estimated for a probability of 0.05 and a confidence level of 0.6 in 16 participants [32]. All statistical analyses were performed with Statistical Package for the Social Sciences (SPSS) version 19.0 for Windows (SPSS Inc., Chicago, IL, USA).

## 3. Results

There were no differences between the beginning of the day in the variables studied in the two days of measurement. Workers started the day in similar conditions. There were no differences in height, weight, and discomfort at the beginning of the day for the control and intervention day. Assembly line workers reduced their height and body weight significantly on the control day and on the intervention day (Table 1). No differences between the spinal shrinkage of both days were found. Spinal sagittal alignment did not change at the end of the control working day in the thoracic and lumbar spine. On the intervention day, a significant increase of lumbar lordosis degrees was observed. There was no significant increase in thoracic kyphosis degrees. Pre- and post-testing lower and upper back discomfort were equal to zero on both days. 

On the other hand, a large effect of the intervention on the difference between post-and pretesting of the thoracic angle was observed. There were no significant main effects (*p* > 0.05), apart from a thoracic angle.

## 4. Discussion

The height and body weight of the workers decreased significantly on the control and intervention day. Upper and lower back discomforts were not modified on both days’ analyses. Thoracic kyphosis was not significantly modified on both days, while lumbar lordosis was significantly increased on the day of the intervention day. Using a spinal decompressor during two breaks in the workday does not seem to be effective to prevent spinal shrinkage caused by prolonged standing work in assembly line workers. 

Assembly line workers lost 1.33 cm in height on the control day. This change throughout the working day (8 h) was significant. Spinal shrinkage observed in the present study was similar to other studies in prolonged standing workers [6,7]. Spinal shrinkage was greater in physically demanding standing jobs that contain load handling than in lighter or sitting jobs [33,34]. When the spinal traction device was used, the loss of worker’s height was lower than on the control day, but no differences were observed between the spinal shrinkage between both days. Future studies could determine if the use of a lumbar traction device in a higher dose, or more days per week, could generate significant changes in relation to the control day.

The values found for the thoracic and lumbar angles at the beginning of the day were similar to those described in the assembly line working population of a manufacturing company [7]. The values in the thoracic area (49.75°) are higher than those found by Muyor et al. in a horticultural company (32.7°) [18]. This difference could be because the study was only analyzed in female workers. Some authors have found differences between gender in spinal sagittal alignment [35,36,37,38].

In the present study, assembly line workers increased thoracic and lumbar curves at the end of the workday, but this change was not significant on the control day. This tendency was observed in a previous study of assembly line workers, in which thoracic kyphosis and lumbar lordosis increased significantly at the end of the day [7]. In the current study, only workers without discomfort were included, while in the previous study, the sample comprised workers with and without upper and lower back discomfort. Contradictory results can be found in other working populations. In a hospital working population, no changes in spinal sagittal alignment were observed during a workday [39].

On the control day, a nonsignificant increase in thoracic and lumbar curvature was observed, while on the intervention day, only lumbar lordosis increased significantly, keeping the thoracic angle unchanged. In previous studies, it has been observed that the minimum clinically relevant change for the modification of the lumbar angle in subjects with “spinal deformity” was 6° [40]. One of the explanations for this increase in lumbar lordosis could be by stretching and elongation of the hamstrings and erectors spinae muscles. This could occur due to the position used during the intervention with the spinal traction device, in which the hip is in flexion and the knees slightly bent, producing a muscular stretching (Figure 1). Diab and Mustafa observed an increase in lumbar lordosis (+6.2°) in people with low back pain, in an intervention for 10 weeks, 3 times a week, with lumbar extension traction (15 min) and stretching of the hamstring and erector spinae (3 × 30 s holding) [13]. The change in the adaptation of the sagittal alignment of the spine throughout a prolonged standing working day could be due to the use of the spinal traction device, but future research carried out with larger samples should confirm these findings.

The normal values of thoracic kyphosis are 40–45°, depending on the authors [41,42,43]. Assembly line workers showed an average value of 50.46° (±5.16) at the beginning of the workday, referred to as hyperkyphosis. Hyperkyphosis has been associated with low values of hamstring flexibility and a lack of abdominal and paravertebral strengthening [41]. Thoracic kyphosis did not change on the intervention day (−0.06° ± 1.7), although on the control day increased non significantly (+1.01° ± 2.39). The analysis of the effect size showed a trend in which the spinal traction intervention prevented the increase of the thoracic angle during the workday. These findings should be interpreted with caution due to the small sample size.

In the present study, levels of upper and lower back discomfort were equal to zero at the beginning and the end of both days. For this reason, it was not possible to know if there is an effect on the precept of the appearance of discomfort with the use of the device. The utilization of the traction device did not increase the discomfort among the participants. In previous research, assembly line workers reported increased lumbar discomfort at the end of the day. Traction interventions such as treatment of low back pain seem to have limited or no impact on these clinical outcomes studied in previous studies [22]. Prasad et al. observed that intervention with inversion traction used intermittently with a physiotherapist significantly showed no differences in the self-perception of low back pain, but it reduced the need for surgery in people with hernia and low back pain [24]. Despite this finding, the effectiveness of this treatment is unclear, and more studies are needed. Different types of traction (inversion, mechanic, auto-traction, aquatic), and different traction parameters are hardly comparable due to heterogeneity [22]. The objectives of future studies should be discovering optimal dosage and traction parameters to inform of standardized and effective traction protocols. From the point of view of the researchers in this investigation, the use of this device appears to be safe and does not increase back discomfort. In this research, the feasibility of applying a spinal traction device in the workplace to modify the spinal shrinkage throughout the working day to reduce the compressive load of the spine was studied.

Recruitment of the sample was small. At a future clinical study, the sample will be increased to include women as well. The acute effect of a decompressor intervention was only studied during one day. Further research is necessary to analyze its long-term efficacy in the treatment of spinal shrinkage. Participants without discomfort were a limitation of the study. It would be interesting to carry out a study with a population who develop discomfort or low back pain during the workday to assess its effectiveness in reducing discomfort. The traction device utilized in the present study does not perform inversion traction. For this reason, its use is easier, but the traction dosage may not represent an effective dose, given that it has been evaluated only for one day and with limited use in a short time. Traction of less than 25% of body weight has been described as a low dose [42]. The use of this type of traction makes it easy for workers to use it comfortably, but the traction force is unknown and may vary between participants. In future research, it would be interesting to assess the prolonged use of this spinal traction device in the work environment.

## 5. Conclusions

Use of a spinal traction device, during 3 min in two breaks in the workday, does not significantly reduce the spinal shrinkage of healthy workers. Lumbar lordosis angle increased significantly at the end of the spinal traction intervention day. The use of the traction device did not increase the discomfort in the participants. Using the device is feasible during the workday on an assembly line. Prospective studies would be necessary to clarify the possible benefits of the traction device.

## Figures and Tables

**Table 1:** Characteristics and differences post–pre workday of the participants (*N* = 16).

Outcome	Control Day		Intervention Day				
	Pre	Post–Pre	Pre	Post–Pre	*F*	*p*	η^2^
Height (cm), Mean (SD)	175.93 (3.78)	−1.33 (0.72) ^a^	175.64 (3.77)	−0.99 (0.23) ^a^	1.34	0.263	0.073
Body weight (kg), Mean (SD)	80.4 (10.06)	−0.35 (0.58) ^a^	80.28 (10.1)	−0.46 (0.43) ^a^	1.11	0.307	0.061
Thoracic angle (°), Mean (SD)	50.46 (5.16)	+1.01 (2.39)	49.66 (8.67)	−0.06 (1.7)	6.35	0.022	0.272
Lumbar angle (°), Mean (SD)	21.86 (7.93)	+1.33 (3.08)	22.86 (7.26)	+1.86 (2.53) ^a^	0.168	0.687	0.010

^a^: *p* ≤ 0.05 Significant differences between pre and post day. *F*: *F* value, η^2^: eta-squared.

## Data Availability

The data are not publicly available due to privacy policy on participant data.
